# Caspase-12 Is Present During Craniofacial Development and Participates in Regulation of Osteogenic Markers

**DOI:** 10.3389/fcell.2020.589136

**Published:** 2020-10-15

**Authors:** Barbora Vesela, Adela Kratochvilova, Eva Svandova, Petr Benes, Kamila Rihova, Anne Poliard, Eva Matalova

**Affiliations:** ^1^Laboratory of Odontogenesis and Osteogenesis, Institute of Animal Physiology and Genetics, Academy of Sciences, Brno, Czechia; ^2^Department of Experimental Biology, Faculty of Science, Masaryk University, Brno, Czechia; ^3^Laboratory of Orofacial Pathologies, Imaging and Biotherapies, UFR Odontology Montrouge, Paris University, Paris, France; ^4^Department of Physiology, Faculty of Veterinary Medicine, University of Veterinary and Pharmaceutical Sciences, Brno, Czechia

**Keywords:** caspase-12, bone, osteoblast, differentiation, alkaline phosphatase

## Abstract

Caspases are evolutionary conserved proteases traditionally known as participating in apoptosis and inflammation but recently discovered also in association with other processes such as proliferation or differentiation. This investigation focuses on caspase-12, ranked among inflammatory caspases but displaying other, not yet defined functions. A screening analysis pointed to statistically significant (*P* < 0.001) increase in expression of caspase-12 in a decisive period of mandibular bone formation when the original mesenchymal condensation turns into vascularized bone tissue. Immunofluorescence analysis confirmed the presence of caspase-12 protein in osteoblasts. Therefore, the osteoblastic cell line MC3T3-E1 was challenged to investigate any impact of caspase-12 on the osteogenic pathways. Pharmacological inhibition of caspase-12 in MC3T3-E1 cells caused a statistically significant decrease in expression of some major osteogenic genes, including those for alkaline phosphatase, osteocalcin and Phex. This downregulation was further confirmed by an alkaline phosphatase activity assay and by a siRNA inhibition approach. Altogether, this study demonstrates caspase-12 expression and points to its unknown physiological engagement in bone cells during the course of craniofacial development.

## Introduction

Caspases are evolutionary conserved proteases traditionally associated with apoptosis and inflammation (Van Opdenbosch and Lamkanfi, 2019). Recently, particular attention has been paid to their novel roles in other processes, such as differentiation, proliferation or autophagy (Shalini et al., 2015; Nakajima and Kuranaga, 2017; Tsapras and Nezis, 2017). The most common investigated caspases include apical proapoptotic caspase-8 and caspase-9, pro-inflammatory caspase-1, and the executive caspases-3 and -7, while others are rather neglected.

Caspase-12 is usually ranked as an inflammatory caspase (Bolívar et al., 2019), however, its exact effect remains unknown. Early studies have suggested the participation of caspase-12 in endoplasmic reticulum stress-induced apoptosis (Nakagawa et al., 2000; Shiraishi et al., 2006). Later, characterization of *Casp12*^–/–^ deficient mice described a suppressive effect of caspase-12 on caspase-1, resulting in an enhanced vulnerability to sepsis (Saleh et al., 2006). Recently, these apoptotic or septic-related roles were not confirmed and its functions remain enigmatic (Salvamoser et al., 2019). Caspase-12 expression was previously described in several tissues (Nakagawa and Yuan, 2000; Kalai et al., 2003; Veselá and Matalová, 2015) in relation to development.

The mandibular bone develops from a mesenchymal condensation where osteoblasts differentiate directly from mesenchymal progenitor cells through an intramembranous process, as most craniofacial bones. In the mouse, a vascularized bone containing all three basic cell types (osteoblasts, osteocytes, osteoclasts) begins to form only 2 days after condensation is achieved. This short but critical period is accompanied by dynamic changes in expression of several specific genes as demonstrated recently for osteogenesis and vascularization synchrony (Veselá et al., 2019). Pilot data (presented here) pointed to a significant increase in caspase-12 expression within this period of mandibular bone development.

The purpose of this investigation was to develop this initial finding and to study caspase-12 in the context of mandibular development. The spatio-temporal analysis was performed to investigate caspase-12 expression pattern in forming mandible with a special focus on bone. Since caspase-12 was strongly present in osteoblasts and since a strong impact on osteoblastic differentiation was demonstrated after general inhibition of the caspases (Mogi and Togari, 2003; Kratochvílová et al., 2020), our aim was to determine whether caspase-12 participates in modulation of the osteogenic pathways.

## Materials and Methods

### Samples

Mice (strain CD1) were purchased from the Laboratory Animal Breeding and Experimental Facility, Masaryk University Brno and kept in the facilities of the Institute of Animal Physiology and Genetics, Czech Academy of Sciences, Czech Republic. Mouse heads were taken as fresh *post-mortem* samples. Embryonic (E) and postnatal (P) stages E13, E15, E18, P1, and P12 were examined. For PCR Array, fresh mandibles at stages E13 and E15 were dissected and cut into 250 μm slices using a McIlwain tissue chopper as described previously (Minaříková et al., 2015). Seven mandibles were used as one biological sample. Tissue slices with region of interest (mandibular bone surrounding the first mouse molar) were selected and the mandibular bone was separated under a stereoscope. The samples were lysed by RLT buffer (Qiagen) for RNA isolation. For immunofluorescence, samples were fixed in 4% paraformaldehyde, dehydrated (ethanol series), treated with xylene, and embedded in paraffin.

### Cell Cultures

A cell line of osteoblastic precursors, MC3T3-E1, was purchased from the European Collection of Cell Culture (ECACC 99072810) and differentiated for 21 days as described previously (Kratochvílová et al., 2020). Growth medium was composed of MEM Alpha (Gibco, United States), 10% fetal bovine serum (FBS), penicillin/streptomycin (1,000 U/ml, 100 μg/ml). Osteogenic differentiation was performed in the same medium containing β-glycerolphosphate (10 mM) and ascorbic acid (50 μg/ml). Medium was changed every second day. During differentiation process, cells were passaged on average once a week to avoid the deposition of thick layer of extracellular matrix which prevents efficiency of cell treatments. Cells differentiated in this way created and deposited cell matrix, expressed osteogenic markers at high levels and were prepared for caspase-12 downregulation. MC3T3-E1 cells were examined before differentiation and then every 7 days to measure levels of caspase-12 expression. After 3 weeks of differentiation, cells were seeded at a density of 5,000 cell/cm^2^. Caspase-12 Inhibitor Z-ATAD-FMK (FMK013, R&D Systems) was used for inhibition of caspase-12 proteolytic activity in cell culture. It was dissolved in DMSO, and applied at a final concentration of 100 μM as recommended by the manufacturer. The control group was treated with the same concentration of DMSO as used for the experimental samples to exclude any side effect of this solvent. Cells were harvested after 6 days of inhibition (protocols previously published e.g., in Kratochvílová et al., 2020) and prepared for RNA isolation, western blot, histological staining, and immunofluorescence.

### RNA Isolation, PCR Arrays, Real-Time PCR

RNA was isolated by RNeasy Mini Kit (Qiagen), then mRNA was transcribed into cDNA using SuperScript VILO (Invitrogen). The Apoptosis PCR Array (PAMM-012Z, Qiagen) was used for analysis of gene expression in developing mandibular bone while the Osteogenesis PCR Array (Qiagen, PAMM-024Z) was used for analysis of osteogenic markers in MC3T3-E1 inhibited by caspase-12 inhibitor. The panel of housekeeping genes included: Actb, B2m, Gapdh, Gusb, and Hsp90ab1. The PCR Array format also included positive and negative controls. *N* = 3 in each group.

Real-time PCR was performed in 10 μl of a final reaction mixture containing the one-step GB Ideal PCR Master Mix (Generi Biotech). Alkaline phosphatase (Mouse *Alpl*, Mm00475834_m1), Osteocalcin (Mouse *Bglap*, Mm03413826_mH), Phex (Mouse *Phex*, Mm00448119_m1) and Dentin matrix protein 1 (Mouse *Dmp1*, Mm01208363_m1) expression was detected by using a TaqMan Gene Expression Assay (Thermo Fisher Scientific) with normalization based on actin levels (Mouse Actb, Mm02619580_g1).

### Immunofluorescence

Frontal sections (5 μm) of mouse heads were used for immunofluorescent detection. Histological sections were de-paraffinized in xylene and rehydrated in a gradient series of ethanol, finishing in water. Sections were pre-treated in citrate buffer (98°C/10 min) for antigen retrieval and then incubated with primary Anti-Caspase-12 antibody (2202, Cell Signaling, Danvers, MA) overnight. For immunofluorescence, cells grown on glass were fixed, pre-treated with 0.1% triton and incubated with the primary antibodies for caspase-12 (see above) or osteocalcin (ab93876, Abcam). Primary antibodies were followed by incubation with secondary anti-rabbit antibody Alexa Fluor 488 (Thermo Fisher Scientific, United States) (1:200) for 40 min at RT. Nuclei were detected by ProLong Gold Antifade reagent with DAPI (Thermo Fisher Scientific, United States). For immunohistochemistry, mandibular slices were treated as described above. Incubation with primary antibodies: osteocalcin, caspase-12 and Runx2 (sc10758, Santa Cruz Biotechnology) was followed by treatment with the peroxidase-conjugated streptavidin-biotin system (Vectastain) and the chromogen substrate diaminobenzidine (DAB, K3466; Dako). Slides were counterstained with hematoxylin. Negative and positive controls for Caspase-12 primary antibody are included in Supplementary Figure 1.

### Western Blot

MC3T3-E1 cells were harvested and lysed by boiling in sodium dodecyl sulfate (SDS)-loading buffer containing 0.1 M Tris (pH 6.8), 16% v/v glycerol, 3.2% w/v SDS, 10% v/v β-mercaptoethanol and 0.005% w/v bromophenol blue. Cell lysates were subjected to sodium dodecyl sulfate polyacrylamide gel electrophoresis (SDS-PAGE) and electroblotted to polyvinylidene fluoride (PVDF) membrane (Millipore). The blot was probed with anti-Caspase-12 Antibody (2202, Cell Signaling) and developed with anti-rabbit IgG secondary antibody conjugated with horseradish peroxidase (A4914, Sigma-Aldrich, St. Louis, MI) by standard procedure using Clarity Max Western ECL Substrate (Biorad).

### siRNA Gene Silencing

Differentiated MC3T3-E1 cells were seeded at density 7,000 cells/cm^2^. Next day cells were transfected with 20 nM Silencer Select pre-designed siRNA *Casp12* (ID: s63375, Catalog No. 4390771, Ambion) using Lipofectamine RNAiMAX Reagent (13778, Life Technologies) according to the producer’s instructions. Silencer Select Negative Control No. 1 siRNA (Catalog No. 4390843, Ambion) was used as a control. Cells were treated for 3 days; the medium with siRNA-Lipofectamine complexes was changed after 48 h of cultivation. *N* = 4 in each group.

### Staining for Alkaline Phosphatase Activity

Fixed cells were stained with 300 μl of Fast blue mixture containing 4 mg of naphthol AS-TR phosphate disodium salt (Sigma, N6125) in 150 μl of N, N-dimethylformamide (Fluka, 40248) and 12 mg of Fast blue BB Salt hemi(zinc chloride) salt (Sigma, F3378) in 15 ml of 0.1 M Tris-HCl buffer (pH 9.6) for 10 min in the dark. *N* = 3 in each group.

### Statistical Analysis

PCR Arrays data were statistically evaluated by Qiagen Gene Globe as recommended by the manufacturer (available on-line). Statistical significance was determined as *P* < 0.05, the threshold of fold regulation as ±2. Three biological replicates were evaluated in each group. Real-time PCR expression levels were calculated using the ΔΔCT method and results were analyzed using two-tailed *t*-test.

## Results

### Caspase-12 Is Among Apoptosis-Related Genes Expressed During Mandibular Bone Development

**FIGURE 1:**
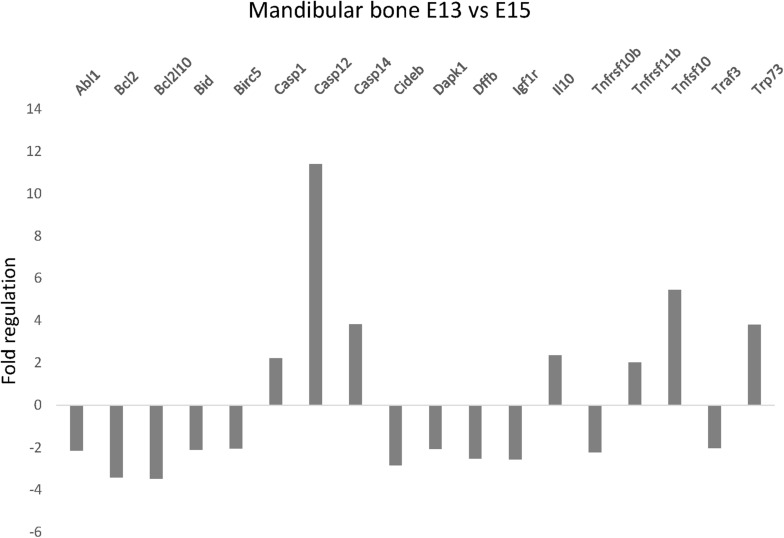
Apoptosis-related gene expression in the developing mandible. Changes in expression of apoptosis-related genes in developing mandibular bone in the mouse. Analysis shows fold regulations (–2/+2 is used as the threshold) of gene expression between the prenatal/embryonic (E) days 13 and 15 detected by PCR Array. Statistically significant (*P* < 0.05) changes are displayed.

To evaluate apoptosis-related gene expression during mouse mandibular bone development, apoptotic PCR Array was used. Among the 84 tested genes, 7 were significantly increased and 11 decreased more than 2-fold between prenatal stages E13 and E15 (Figure 1). The most prominent change in gene expression was detected for caspase-12 (11.4-fold upregulation, *P* < 0.001). Other significantly upregulated genes included caspase-1 (2.26, *P* = 0.0047) caspase-14 (3.83, *P* = 0.0484) Il10 (2.37, *P* = 0.0216), Tnfrsf11b (gene for osteoprotegerin; 2.03, *P* = 0.0062), Tnfsf10 (gene for Trail; 5.45, *P* < 0.001), and Trp73 (3.81, *P* = 0.0486) while Abl1 (−2.14, *P* = 0.003), Bcl2 (−3.4, *P* = 0.0034), Bcl2l10 (−3.46, *P* = 0.0403), Bid (−2.09, *P* = 0.0014), Birc5 (−2.03, *P* < 0.001), Cideb (−2.83, *P* = 0.0127) Dapk1 (−2.06, *P* = 0.003), Dffb (−2.5, *P* = 0.0174), Igf1r (−2.55, *P* = 0.0033), Tnfrsf10b (gene for DR5; −2.12, *P* = 0.0026), and Traf3 (−2.02, *P* = 0.0026) were significantly downregulated.

### Caspase-12 Protein Is Present in Developing Mandibular Bone and Adjacent Structures

**FIGURE 2:**
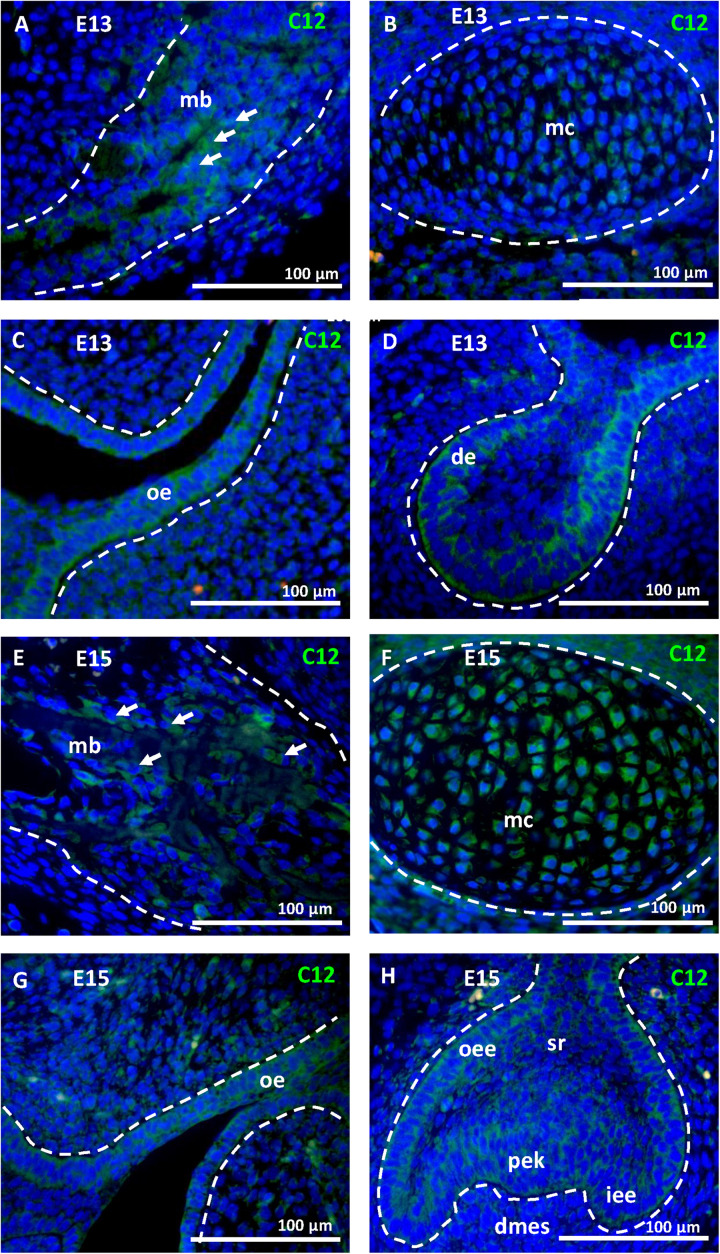
Caspase-12 protein in prenatal development. Immunofluorescence analysis of caspase-12 (green) in developing mandibular bone and adjacent structures of the lower jaw. Caspase-12 at the stage E13 was localized in the mandibular bone **(A)**, Meckel’s cartilage **(B)**, oral epithelium **(C)**, and first lower molar **(D)**. Caspase-12 positivity at the stage E15 was localized in the mandibular bone **(E)**, Meckel’s cartilage **(F)**, oral epithelium **(G)**, and developing first lower molar **(H)**. Scale bar: 100 μm, counterstained by DAPI (blue). Arrows point to osteoblasts. mb, mandibular bone; mc, Meckel’s cartilage; oe, oral epithelium; de, dental epithelium; oee, outer enamel epithelium; dmes, dental mesenchyme; iee, inner enamel epithelium; sr, stellate reticulum; pek, primary enamel knot.

**FIGURE 3:**
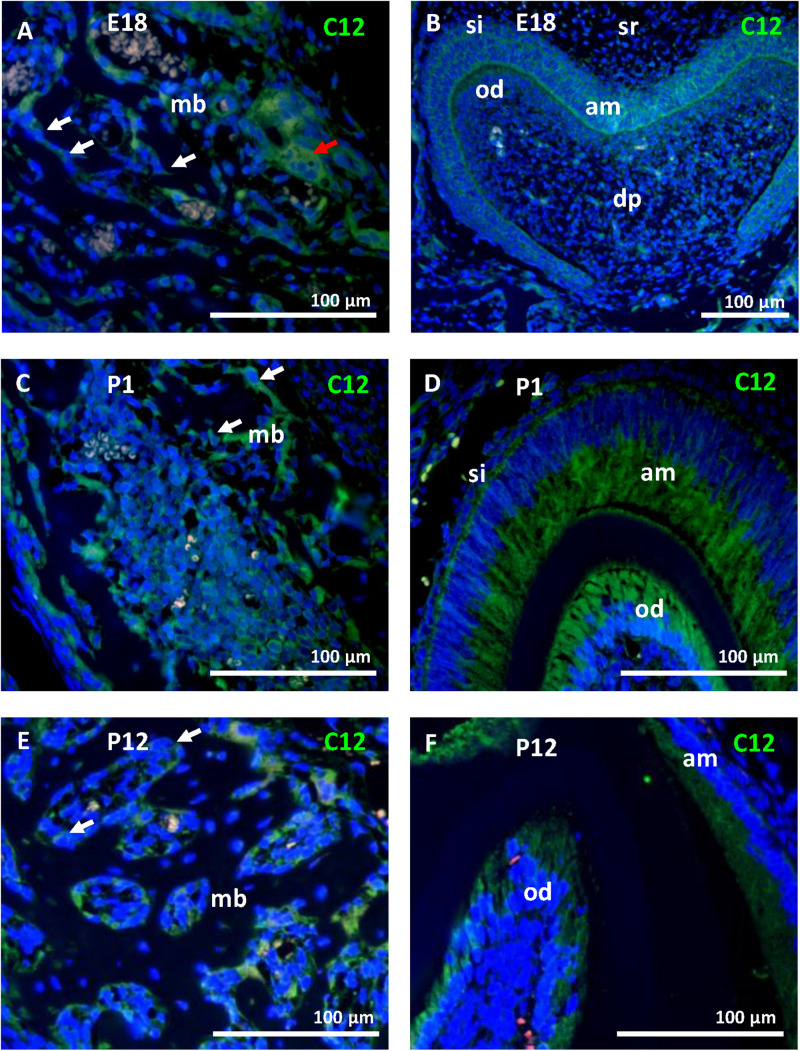
Caspase-12 protein in perinatal and postnatal development. Immunofluorescence analysis of caspase-12 (green) in mandibular bone and the first lower molar at perinatal stages (E18 and P1) and postnatal stage P12. Caspase-12 was localized in the mandibular bone, predominantly in osteoblasts **(A,C,E)** at all of these stages. In the developing tooth, caspase-12 was positive mainly in ameloblasts and odontoblasts **(B,D,F)**. Scale bar: 100 μm, counterstained by DAPI (blue). White arrows point to osteoblasts, a red arrow points to osteoclasts. mb, mandibular bone; si, stratum intermedium; sr, stellate reticulum; dp, dental pulp; am, ameloblasts; od, odontoblasts.

To ascertain the increase in *Casp12* mRNA expression between stages E13 and E15, at the protein level and precise caspase-12 localization, immunofluorescent analyses were performed on the developing mandibular bone at the area surrounding the first molar. At E13, when the mandibular bone starts to develop, caspase-12 protein was observed in the condensed mesenchymal cells (Figure 2A). Moreover, caspase-12 was also detected in adjacent mandibular structures involving the Meckel’s cartilage (Figure 2B), oral epithelium (Figure 2C) and tooth epithelium (Figure 2D). At E15, the mandibular bone contained all three types of bone cells, osteoclast, osteoblasts and osteocytes, and formed calcified extracellular matrix. At this stage, caspase-12 was still expressed in the developing bone, especially by osteoblasts (Figure 2E). Expression of caspase-12 in Meckel’s cartilage was maintained but more pronounced than at E13 (Figure 2F). Oral and tooth epithelium were still positive, moreover some weak signal of caspase-12 was observed in dental pulp (Figures 2G,H). Next, prenatal stage E18 and postnatal stages P1 and P12 were examined to further delineate caspase-12 expression during the mineralized tissue development. At all these stages, caspase-12 was also detected in the mandibular bone, predominantly in osteoblasts (Figures 3A,C,E). In the tooth, caspase-12 was observed in differentiating ameloblasts and odontoblasts with the most evident signal around birth (Figures 3B,D,F).

### Caspase-12 Is Abundantly Present in Osteoblasts

**FIGURE 4:**
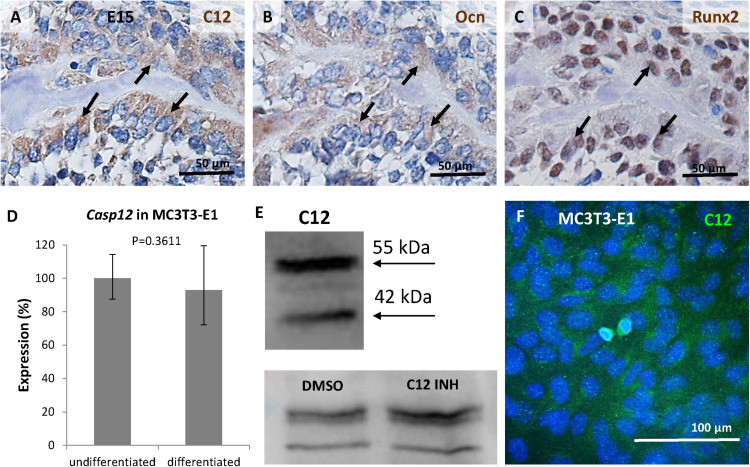
Expression of caspase-12 in osteoblasts. Comparison of serial sections of the mouse mandibular bone at the prenatal/embryonic **(E)** day 15 showing co-localization of caspase-12 **(A)** with osteoblastic markers osteocalcin **(B)** and Runx2 **(C)** within the bone. Positive cells are brown, counterstained by hematoxylin (blue). Arrows point to cells in the same region. Scale bar: 50 μm. Expression of caspase-12 in undifferentiated and differentiated MC3T3-E1 cells **(D)** showing stable expression of *Casp12* after 21 days of differentiation. Western blot analysis **(E)** and immunofluorescence **(F)** confirmed the presence of caspase-12 protein in MC3T3-E1. E: 55 kDa-full length caspase-12, 42 kDa-cleaved caspase-12, DMSO: caspase-12 forms after DMSO treatment, C12 INH: caspase-12 forms after specific inhibitor treatment. F: Caspase-12 in green, DAPI in blue. Scale bar: 100 μm.

To confirm the presence of the caspase-12 protein in osteoblasts *in vivo*, serial sections of mandibular bone at stage E15 were used. Expression of caspase-12 (Figure 4A) overlapped with localization of the osteoblastic markers osteocalcin (Figure 4B) and Runx2 (Figure 4C). To get closer insight into caspase-12 function in osteoblasts, expression of caspase-12 was evaluated in MC3T3-E1, an osteoblast cell line derived from calvaria. *Casp12* mRNA was expressed at a similar level, both in undifferentiated and MC3T3-E1 cells induced to differentiate toward an osteoblast phenotype (Figure 4D). *Casp12* mRNA expression remained stable during all the course of the differentiation process (data not shown). Western blot analysis revealed the presence of both the full length (55 kDa) and cleaved (42 kDa) forms of the caspase-12 protein in MC3T3-E1 cells (Figure 4E). The amount of non-cleaved full length form increased after treatment with caspase-12 inhibitor. Furthermore, caspase-12 protein presence in MC3T3-E1 cells was confirmed by immunofluorescence analysis (Figure 4F).

### Osteoblastic Markers Are Downregulated After Caspase-12 Inhibition

**FIGURE 5:**
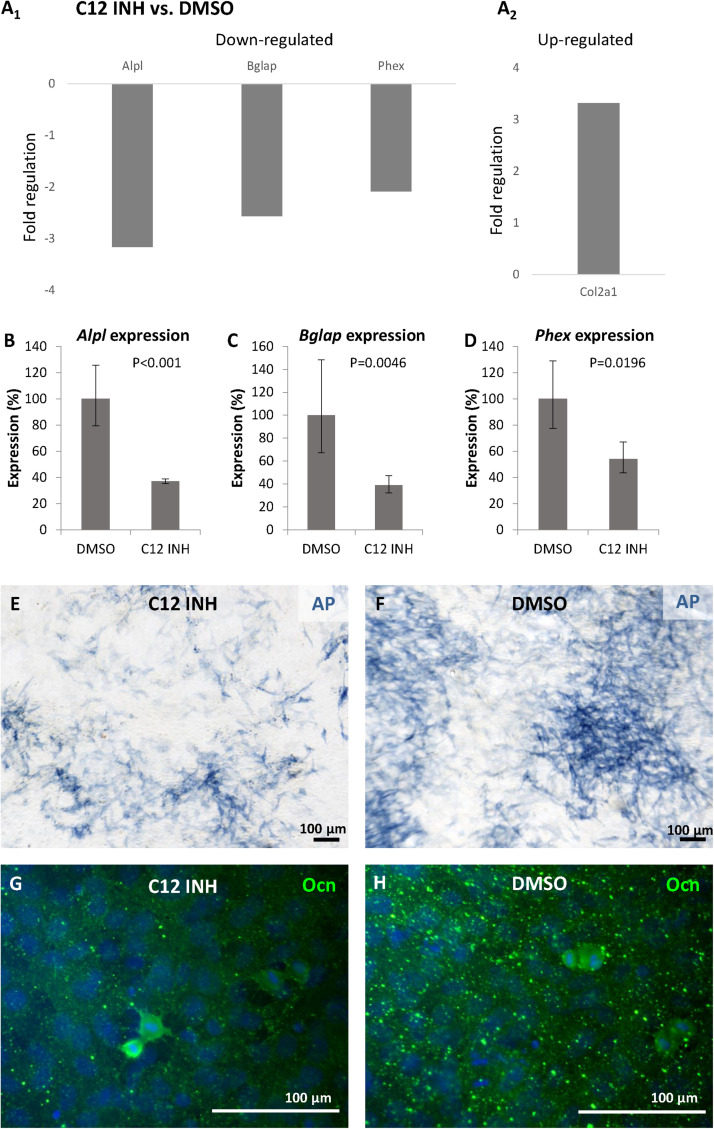
Inhibition of caspase-12 in MC3T3-E1 cells. Changes in gene expression of osteogenesis-related genes **(A_1_,_2_)** in differentiated MC3T3-E1 cells after inhibition of caspase-12 by the specific inhibitor Z-ATAD-FMK, and DMSO-treated cells used as a control. Analysis shows fold regulations (–2/+2 is used as the threshold) of gene expression, statistically significant (*P* < 0.05) changes are displayed. Real-time PCR confirmed statistically significant decrease of *Alpl* **(B)**, *Bglap* **(C)**, and *Phex* **(D)** in inhibited MC3T3-E1. Results are presented as means ± standard deviations and were analyzed using two-tailed *t*-test. Staining for activation of alkaline phosphatase (blue) in MC3T3-E1 cells after caspase-12 inhibition **(E)** compared to control **(F)**. Immunofluorescence staining of osteocalcin expression (green) in differentiated MC3T3-E1 after inhibition of caspase-12 **(G)** compared to control **(H)**. Scale bar: 100 μm, counterstained by DAPI (blue).

As caspase-12 is produced by osteoblastic cells, its possible role was explored through inhibition studies. Differentiated MC3T3-E1 were treated for 6 days with the caspase-12 Inhibitor Z-ATAD-FMK and an Osteogenic PCR Array was performed. Analysis revealed a statistically significant downregulation in expression of 3 important osteogenic genes (Figure 5A1): *Alpl* (alkaline phosphatase) was decreased more than 3-times, *Bglap* (osteocalcin) decreased more than 2.5-times and expression of *Phex* (Phosphate-regulating neutral endopeptidase, X-linked) decreased more than 2-times. Downregulation of these genes was further confirmed by real-time PCR. *Alpl* expression (Figure 5B) decreased to 37% compared to control (*P* < 0.001), *Bglap* expression (Figure 5C) decreased to 39% (*P* = 0.0047) and *Phex* expression (Figure 5D) decreased to 54% (*P* = 0.0196) of the normal value. Only one upregulated gene (*Col2a1*, 3.32-times increased, *P* < 0.001) was detected after caspase-12 inhibition (A_2_). The whole osteogenic panel showing gene expression in individual samples is presented in a heatmap (Supplementary Figure 2) At the protein level, a weaker staining for alkaline phosphatase activity and osteocalcin immunofluorescence were observed in caspase-12 inhibited samples as compared to the controls (respectively, Figures 5E–H).

### Osteoblastic Markers Are Downregulated After *Casp12* Gene Silencing

**FIGURE 6:**
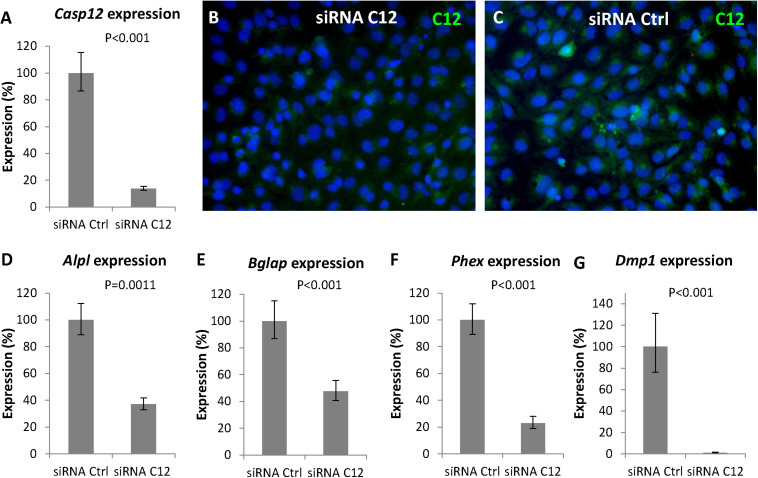
Gene silencing of *Casp12* in MC3T3-E1. Gene silencing of *Casp12* in MC3T3-E1 cells using specific siRNA showed statistically significant reduction of *Casp12* expression **(A)**. Real-time PCR confirmed statistically significant decrease in expression of *Alpl* **(D)**, *Bglap* **(E)**, *Phex* **(F)**, and *Dmp1* **(G)** after *Casp12* silencing. Results are presented as means ± standard deviations and were analyzed using two-tailed *t*-test. Immunofluorescence detection of caspase-12 (green) protein in MC3T3-E1 after Casp12 gene silencing **(B)** compared to control **(C)**, DAPI in blue.

To further confirm the involvement of caspase-12 in the regulation of gene expression of the above mentioned genes, a *Casp12* gene silencing approach was performed. Silencing was efficient since 3 days after transfection of differentiated MC3T3-E1 cells with a specific siRNA, *Casp12* gene expression (Figure 6A) decreased to 14% compared to control (*P* < 0.001). This efficient silencing was confirmed at the protein level (Figures 6B,C). Similarly, as observed with the caspase-12 inhibitor, expression of *Alpl* (Figure 6D), *Bglap* (Figure 6E), and *Phex* (Figure 6F) were significantly downregulated. *Alpl* decreased to 37% (*P* = 0.0011), *Bglap* to 48% (*P* < 0.001), and *Phex* to 23% (*P* < 0.001). In addition, a temporal silencing effect of *Casp12* siRNA, on *Bglap* gene expression was shown (Supplementary Figure 3). To further support the hypothesis on a novel role of caspase-12 in mineralization, expression of *Dmp1*, a critical marker for bone and dentin mineralization, not included in the osteogenic array, was analyzed. Expression of *Dmp1* dropped to 1% (*P* < 0.001) after *Casp12* gene silencing (Figure 6G).

## Discussion

Caspase-12 is a cysteine protease with yet unclear roles supported by often conflicting results. So far, caspase-12 protein was detected in some organs including heart, lungs, nose and hair follicles (Kalai et al., 2003; Veselá and Matalová, 2015). Several caspases were previously reported also in craniofacial bones (Svandova et al., 2018) but caspase-12 was investigated in this context for the first time. The initial examination related to mandibular bone and based on mRNA expression was focused on the period when the bone initially forms from the original mesenchymal condensation. This is essential also for further tooth-bone interactions heading for a dynamic anchorage of the tooth within the jaw in the context of functional dentition. Therefore, the spatio-temporal caspase-12 analysis was focused on the bone but also on the adjacent tooth developing in synchrony.

Since the strongest expression of caspase-12 in bone was identified within osteoblasts, further analysis was focused on this cell population. Caspase-12 was expressed in osteocalcin and Runx2-positive cells *in vivo* and both forms, full length procaspase-12 and cleaved active caspase-12, were identified in MC3T3-E1 cells. This osteoblastic cell line has a calvarial origin and is a valuable *in vitro* model for craniofacial bone research (Sudo et al., 1983). So far, caspase-12 expression in osteoblasts was considered mostly in the context of cell death and pathological conditions. For example, increased activation of caspase-12 was associated with endoplasmic reticulum stress and osteoblast apoptosis caused by fluorosis (Liu et al., 2015). In the present investigation, caspase-12 was observed in osteoblastic cells without a link to apoptosis, therefore other roles of this protease should be considered in the forming bone.

To tackle the question of these possible novel roles of caspase-12 in osteoblasts, functional experiments at the mRNA as well as protein levels were performed using the MC3T3-E1 cells. Notably, pharmacological inhibition of caspase-12 protein caused a significant downregulation of expression of the essential osteoblastic genes *Alpl*, *Bglap* and *Phex*. A similar decrease was previously observed following a general caspase inhibition in MC3T3-E1 cells (Kratochvílová et al., 2020). Therefore, caspase-12 is likely to be at least one of the major contributors to this modulatory effect within the osteogenic pathways. To further support our findings and exclude any non-specificity of the Z-ATAD-FMK inhibitor (Roy et al., 2008), *Casp12* gene silencing, using a specific siRNA, was performed and the significant decrease in all three genes was confirmed.

Despite running attempts to understand the mechanism of the caspase-12 effect in development and homeostasis, little information is yet available (Salvamoser et al., 2019). One of the proposed mechanisms of caspase-12 functions was the recruitment of caspase-1 from its activation complexes and thus negative regulation of the inflammasome, but this function was not confirmed (Saleh et al., 2006; Vande Walle et al., 2016). Notably, in our original PCR Array based panel of pro-apoptotic molecules, caspase-1 expression was elevated along with caspase-12 which suggests an involvement of both caspases. Up to now, interactions between caspase-1 and caspase-12 at the protein level were essentially observed in inflammatory processes (Roy et al., 2008).

One of the caspases known to activate caspase-12 is caspase-7 (Rao et al., 2001). It is also expressed during craniofacial development, particularly in the mandibular bone where its lack causes a significant decrease in adult bone volume (Svandova et al., 2014). So far, caspase-7 and caspase-12 interplay was shown as an important step during neuronal death (De La Cadena et al., 2014). Their co-localization within mandibular osteoblasts raises the possibility that caspase 7 could also participate in the non-apoptotic functions of caspase-12 within the bone.

Due to yet undefined roles and mechanisms related to caspase-12, the position of this caspase within the caspase networks is not yet understood. Based on our findings demonstrating a reduction of alkaline phosphatase activity upon caspase-12 inhibition/silencing and on earlier published data reporting a similar effect after inhibition of caspase-2, −3, and −8 (Mogi and Togari, 2003), caspase-12 could therefore be involved in the same caspase cascade.

Adding to the complexity, inter-species differences in caspase-12 functions are expected based on structural findings and activation processing of this caspase (Roy et al., 2008). Indeed, in humans, most people express a truncated form of caspase-12 lacking the catalytic domain and only about 20% of African descent people express the full length protease known to be a risk factor for developing sepsis (Saleh et al., 2004). In contrast in mice, the full length form is always expressed and can thus undergo proteolytic cleavage (Kalai et al., 2003). Until now, caspase-12 substrates have not been fully defined (Van Opdenbosch and Lamkanfi, 2019). One suggested option is caspase-12 autoprocessing (Roy et al., 2008), which is supported by increased amount of full length protein after caspase-12 inhibition observed also in our study.

Despite the complicated understanding of caspase-12 action mechanisms, the present investigation showed a clear-cut effect of its modulation on expression of important osteogenic genes necessary for proper osteogenic regulation. Caspase-12 deficient bone phenotype has not yet been analyzed but alkaline phosphatase deficiency in mice causes hypophosphatasia and impaired mineralization of bone and cartilage (Fedde et al., 1999), osteocalcin-deficient mice develop higher bone mass phenotype with bone defects (Ducy et al., 1996) and *Phex* mutation is associated with X-linked dominant hypophosphatemic rickets involving impaired bone mineralization (Francis et al., 1997). Therefore, all three caspase-12 regulated markers, alkaline phosphatase, osteocalcin and Phex are molecules associated with the regulation of bone mineralization. In accordance with these data, expression of *Dmp1*, another important factor in bone mineralization (Lorenz-Depiereux et al., 2006), was affected by *Casp12* gene silencing. This finding points to novel functions of caspase-12 in osteoblast differentiation. This conclusion would be in agreement with the latest data coming from analysis of caspase-1/-11/-12 triple mutant mice which did not reveal any critical functions of caspase-12 in apoptosis or inflammatory response (Salvamoser et al., 2019) and other functions thus become highly probable. The present investigation is therefore the first indication of such a novel function in craniofacial bone formation. Further studies should help to unravel the complex interactions between caspase-12 and its partners and understand its apoptosis-independent functions.

## Data Availability Statement

All datasets generated for this study are included in the article/Supplementary Material.

## Ethics Statement

Ethical review and approval was not required for the animal study because the research did not include experiments in living animals, all the samples were obtained post-mortem. According to the recent law in the Czechia (359/2012 Sb.), post-mortem collection of animal samples is not considered as an experiment (paragraph 3, letter t) and thus does not require approval by any specific committee.

## Author Contributions

EM designed the study. BV planned the experiments. BV, AK, ES, and KR performed the experiments. PB and AP analyzed the data. BV, EM, and AP wrote the manuscript. All authors contributed to the article and approved the submitted version.

## Conflict of Interest

The authors declare that the research was conducted in the absence of any commercial or financial relationships that could be construed as a potential conflict of interest.
