# Supplementary material for: Caspase-12 Is Present During Craniofacial Development and Participates in Regulation of Osteogenic Markers
Source: Front Cell Dev Biol. 2020 Oct 15;8:589136. doi: 10.3389/fcell.2020.589136 (PMC7593616; doi:10.3389/fcell.2020.589136)
Supplement: Supplementary file 1 [file Data_Sheet_1.PDF]

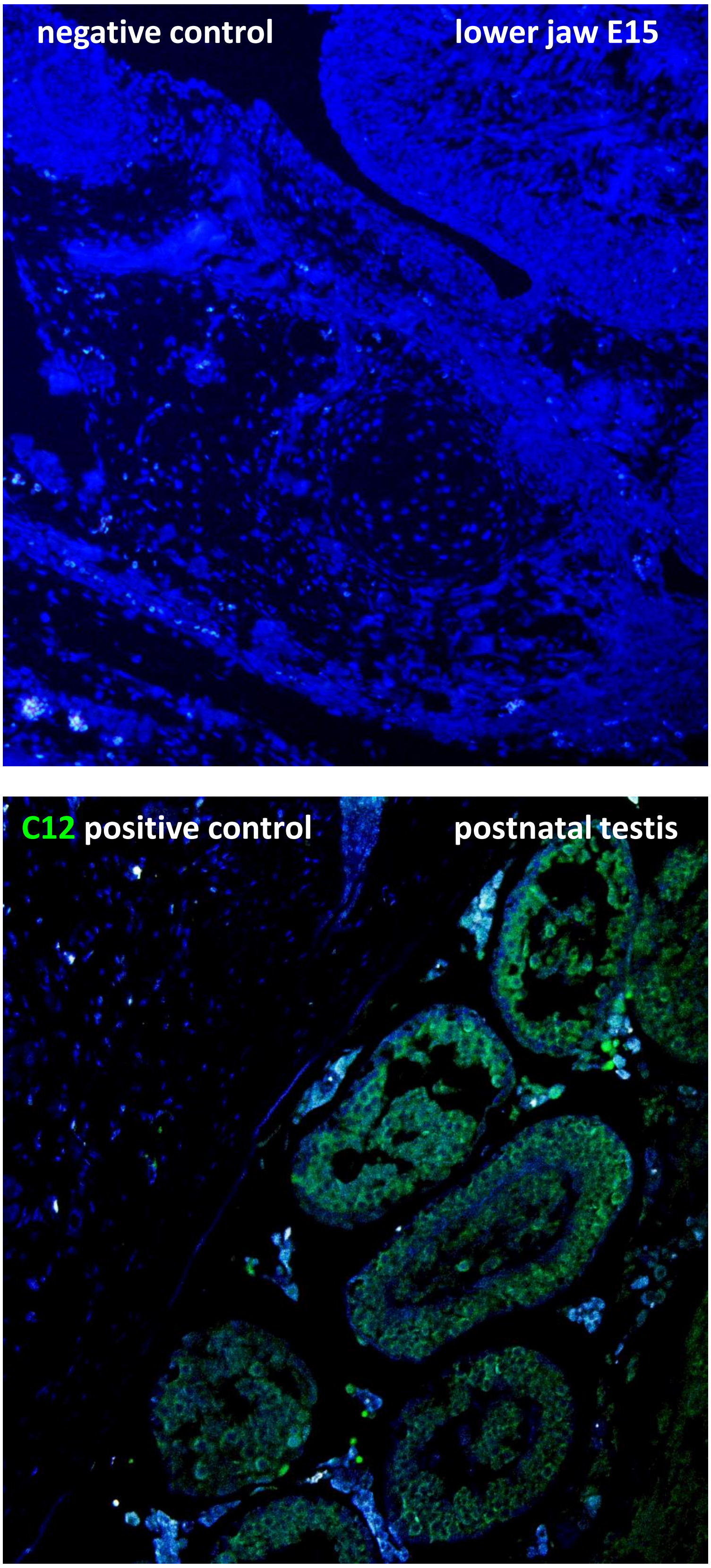

Supplementary figure 1: Immunofluorescent negative control for non-specific binding of secondary antibody showed no signal in mouse lower jaw at stage E15. Positive control for anti-Caspase-12 antibody (2202, Cell Signaling) was shown in mouse testis, the tissue known for caspase-12 expression. Caspase-12 was detected in seminiferous tubules and interstitial tissue, negative staining was observed in surrounding fibrous tissue.

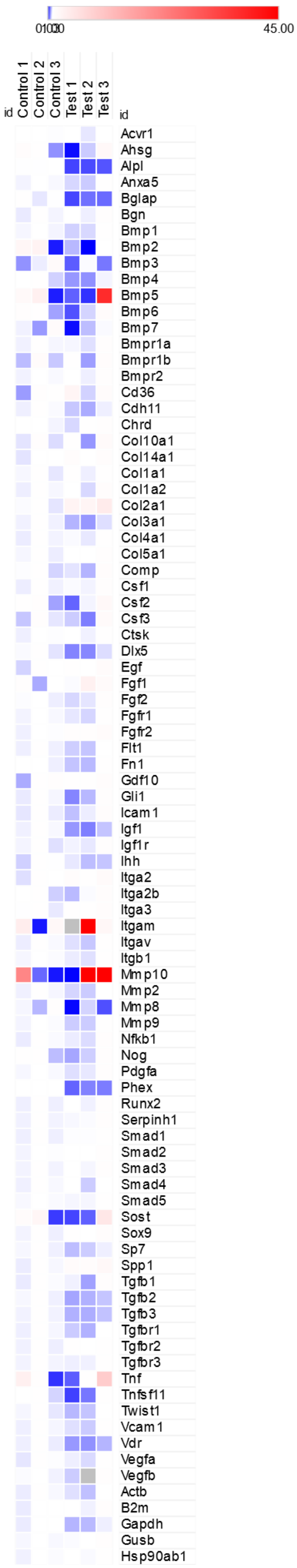

Supplementary figure 2: Heatmap representation of the differences in the osteogenic gene expression between control (DMSO) and test (C12 INH) groups. Colours represent fold change in the gene expression as compared to its mean expression in the control groups. Made in: https://software.broadinstitute.org/morpheus/

Casp12 expression

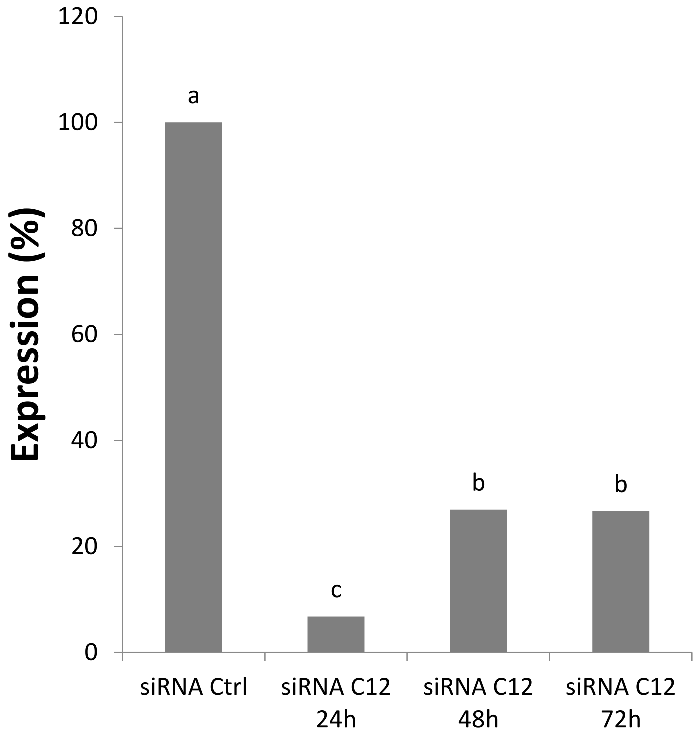

Bglap expression

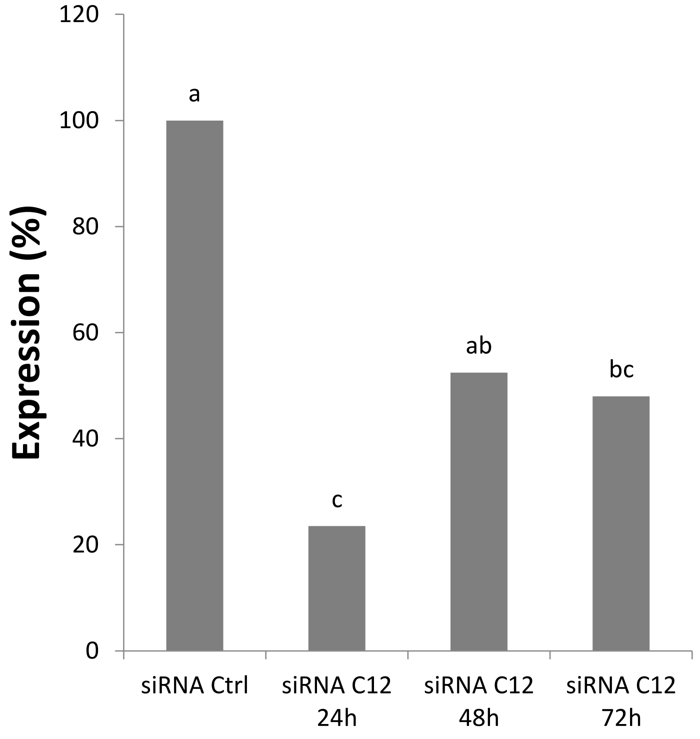

Supplementary figure 3: Temporal dependent regulation of osteocalcin expression (*Bglap*) by *Casp12* silencing. For both genes, there was a statistically significant decrease in expression level between siRNA Control and 24 h and a statistically significant increase between 24 and 48 h of treatment by siRNA c12. The expression of both genes remained unchanged between 48 and 72 h, but for both genes, at 72 h, the expression remained lower than in control samples. P values determined by one-way ANOVA and Tukey HSD post-hoc test. Different letters indicate statistically significant differences.
